# Dipotassium hexa­aqua­nickel(II) bis­[hexa­fluoridozirconate(IV)]

**DOI:** 10.1107/S160053681005350X

**Published:** 2010-12-24

**Authors:** Abdelghani Oudahmane, Noura Mnaouer, Malika El-Ghozzi, Daniel Avignant

**Affiliations:** a Université Blaise Pascal, Laboratoire des Matériaux Inorganiques, UMR CNRS 6002, 24 Avenue des Landais, 63177 Aubière, France

## Abstract

Single crystals of the title compound, K_2_[Ni(H_2_O)_6_][ZrF_6_]_2_, were grown by slow evaporation of a 40% aqueous HF solution in which a stoichiometric mixture of NiCl_2_·6H_2_O, ZrF_4_ and KCl was dissolved. The monoclinic structure is isotypic with its K_2_Cu, K_2_Zn, Cs_2_Zn and Cs_2_Cu analogues. The structure is built up from isolated, slightly elongated octa­hedral [Ni(H_2_O)_6_]^2+^ complex cations (symmetry 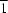) and dimeric [Zr_2_F_12_]^4−^ complex anions (symmetry 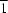) that are also isolated from each other. The [Zr_2_F_12_]^4−^ anion results from the association of two distorted penta­gonal–bipyramidal [ZrF_7_] coordination polyhedra by sharing an equatorial edge passing through an inversion center of the unit cell. Both isolated [Ni(H_2_O)_6_]^2+^ and [Zr_2_F_12_]^4−^ complex ions are situated in planes parallel to (010). They are connected by the eight-coordinated K^+^ ions into a three-dimensional structure. An intricate O—H⋯F hydrogen-bonding network consolidates the structure.

## Related literature

For isotypic structures, see: Fischer & Weiss (1973 ▶); Bukvetskii *et al.* (1993 ▶); Hitchman *et al.* (2002 ▶). For a review on the stereochemistry of zirconium and hafnium fluorido complexes, see: Davidovich (1998 ▶). For background to distortion indices, see: Momma & Izumi (2008 ▶).

## Experimental

### 

#### Crystal data

K_2_[Ni(H_2_O)_6_][ZrF_6_]_2_
*M* *_r_* = 655.45
Monoclinic, 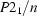

*a* = 6.6090 (1) Å
*b* = 10.0398 (1) Å
*c* = 11.7843 (1) Å
β = 95.897 (1)°
*V* = 777.79 (2) Å^3^
*Z* = 2
Mo *K*α radiation
μ = 3.20 mm^−1^
*T* = 296 K
0.28 × 0.14 × 0.09 mm

#### Data collection

Bruker APEXII CCD diffractometer
Absorption correction: multi-scan (*SADABS*; Bruker, 2008 ▶) *T*_min_ = 0.613, *T*_max_ = 0.748
17267 measured reflections
4436 independent reflections
3858 reflections with *I* > 2σ(*I*)
*R*_int_ = 0.028

#### Refinement

*R*[*F* ^2^ > 2σ(*F* ^2^)] = 0.023
*wR*(*F* ^2^) = 0.052
*S* = 1.06
4436 reflections
131 parameters
All H-atom parameters refined
Δρ_max_ = 0.63 e Å^−3^
Δρ_min_ = −0.57 e Å^−3^

### 

Data collection: *APEX2* (Bruker, 2008 ▶); cell refinement: *SAINT* (Bruker, 2008 ▶); data reduction: *SAINT*; program(s) used to solve structure: *SHELXS97* (Sheldrick, 2008 ▶); program(s) used to refine structure: *SHELXL97* (Sheldrick, 2008 ▶); molecular graphics: *DIAMOND* (Brandenburg, 1999 ▶) and *ORTEP-3 for Windows* (Farrugia, 1997 ▶); software used to prepare material for publication: *SHELXTL* (Sheldrick, 2008 ▶).

## Supplementary Material

Crystal structure: contains datablocks I, global. DOI: 10.1107/S160053681005350X/wm2439sup1.cif

Structure factors: contains datablocks I. DOI: 10.1107/S160053681005350X/wm2439Isup2.hkl

Additional supplementary materials:  crystallographic information; 3D view; checkCIF report

## Figures and Tables

**Table 1:** Hydrogen-bond geometry (Å, °)

*D*—H⋯*A*	*D*—H	H⋯*A*	*D*⋯*A*	*D*—H⋯*A*
O1—H11⋯F1^i^	0.79 (3)	2.94 (2)	3.3300 (14)	112.8 (19)
O1—H11⋯F2^ii^	0.79 (3)	1.92 (3)	2.6877 (14)	164 (2)
O1—H12⋯F4^iii^	0.82 (3)	1.82 (3)	2.6375 (13)	177 (2)
O1—H12⋯F5^iv^	0.82 (3)	2.61 (2)	3.0526 (13)	116 (2)
O2—H21⋯F1^i^	0.78 (2)	2.59 (2)	3.0048 (13)	115 (2)
O2—H21⋯F2^ii^	0.78 (2)	1.95 (2)	2.7227 (13)	167 (2)
O2—H22⋯F4^v^	0.82 (2)	1.92 (2)	2.7362 (13)	173 (2)
O2—H22⋯F6^vi^	0.82 (2)	2.76 (2)	3.1924 (14)	114.9 (17)
O3—H31⋯F1^vii^	0.80 (3)	2.86 (3)	3.2552 (16)	113.3 (19)
O3—H31⋯F5^viii^	0.80 (3)	1.92 (3)	2.7019 (14)	168 (2)
O3—H32⋯F3^iv^	0.80 (3)	3.00 (2)	3.4527 (16)	119 (2)
O3—H32⋯F6^ix^	0.80 (3)	1.97 (3)	2.7427 (15)	163 (2)

Symmetry codes: (i) 
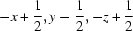
; (ii) 
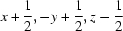
; (iii) 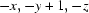; (iv) 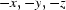; (v) 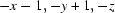; (vi) 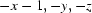; (vii) 
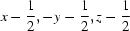
; (viii) 
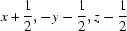
; (ix) 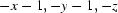.

**Table 2:** Comparison of the geometrical characteristics of the coordination polyhedra in isotypic *M* ^I^_2_[*M* ^II^(H_2_O)_6_][ZrF_6_]_2_ structures determined from single-crystal data (Å, °, Å^3^)

	K_2_[Ni(H_2_O)_6_][ZrF_6_]_2_*^*a*^*	K_2_[Cu(H_2_O)_6_][ZrF_6_]_2_*^*b*^*	K_2_[Zn(H_2_O)_6_][ZrF_6_]_2_*^*c*^*	Cs_2_[Zn(H_2_O)_6_][ZrF_6_]_2_*^*d*^*
Space group	*P*2_1_/*n*	*P*2_1_/*c*	*P*2_1_/*c*	*P*2_1_/*n*
*a*	6.6090 (1)	6.631 (6)	6.631 (1)	6.970 (1)
*b*	10.0398 (1)	9.981 (10)	10.071 (1)	10.515 (2)
*c*	11.7843 (1)	12.921 (12)	12.952 (1)	11.803 (2)
*β*	95.897 (1)	114.20 (15)	114.96 (2)	93.56 (3)
*V*	777.786 (16)	780.01 (1)	784.16 (2)	863.4 (3)
	Ni—O1 = 2.0548 (10) (2×)	Cu—O1 = 1.966 (4) (2×)	Zn—O1 = 2.0856 (2) (2×)	Zn—O3 = 2.096 (6) (2×)
Distances *M*^II^—O	Ni—O3 = 2.0570 (11) (2×)	Cu—O2 = 2.025 (6) (2×)	Zn—O2 = 2.0940 (1) (2×)	Zn—O1 = 2.099 (5) (2×)
	Ni—O2 = 2.0781 (9) (2×)	Cu—O3 = 2.327 (5) (2×)	Zn—O3 = 2.1185 (2) (2×)	Zn—O2 = 2.105 (5) (2×)
Average *M*^II^—O bond length	2.063	2.106	2.099	2.100
Polyhedral volume	11.684	12.335	12.318	12.341
Distortion index (bond length)	0.00483	0.06089	0.00607	0.00156
Quadratic elongation	1.0013	1.0124	1.0011	1.0006
	Zr—F6 = 1.9718 (9)	Zr—F3 = 1.968 (5)	Zr—F3 = 1.9727 (3)	Zr—F3 = 1.962 (5)
	Zr—F5 = 2.0006 (8)	Zr—F1 = 2.004 (5)	Zr—F1 = 2.0018 (3)	Zr—F6 = 1.977 (5)
	Zr—F3 = 2.0293 (9)	Zr—F5 = 2.029 (4)	Zr—F5 = 2.0277 (3)	Zr—F5 = 2.037 (5)
Distances Zr—F (Å)	Zr—F2 = 2.0554 (8)	Zr—F6 = 2.059 (4)	Zr—F6 = 2.0570 (3)	Zr—F4 = 2.067 (4)
	Zr—F1 = 2.0708 (8)	Zr—F2 = 2.063 (4)	Zr—F2 = 2.0668 (4)	Zr—F2 = 2.069 (4)
	Zr—F4 = 2.1468 (9)	Zr—F4 = 2.156 (5)	Zr—F4 = 2.1501 (4)	Zr—F1 = 2.156 (4)
	Zr—F4 = 2.1614 (8)	Zr—F4 = 2.160 (4)	Zr—F4 = 2.1628 (4)	Zr—F1 = 2.180 (4)
Average Zr—F bond length	2.062	2.063	2.063	2.064
Polyhedral volume	13.669	13.674	13.675	13.692
Distortion index (bond length)	0.02662	0.02650	0.02654	0.02985
	K—F5 = 2.6496 (10)	K—F1 = 2.668 (5)	K—F1 = 2.6506 (3)	Cs—F6 = 2.911 (5)
	K—F3 = 2.7366 (9)	K—F6 = 2.750 (6)	K—F2 = 2.7395 (4)	Cs—F4 = 3.046 (4)
	K—F6 = 2.7603 (11)	K—F3 = 2.756 (5)	K—F5 = 2.7633 (2)	Cs—F2 = 3.057 (4)
Distances K—F/O	K—F2 = 2.7658 (9)	K—F5 = 2.767 (5)	K—F6 = 2.7739 (2)	Cs—F3 = 3.065 (5)
	K—F1 = 2.7895 (9)	K—F2 = 2.799 (5)	K—F3 = 2.8094 (2)	Cs—F3 = 3.102 (5)
	K—O2 = 2.8927 (10)	K—O3 = 2.942 (5)	K—O1 = 2.8968 (3)	Cs—O2 = 3.218 (5)
	K—O1 = 3.1012 (10)	K—O1 = 2.980 (5)	K—O2 = 3.0873 (5)	Cs—O1 = 3.236 (5)
	K—F6 = 3.1684 (12)	K—F3 = 3.1307 (7)	K—F3 = 3.1707 (7)	Cs—F5 = 3.228 (6)
Average *M*^I^—F/O bond length	2.858	2.849	2.861	3.118
Polyhedral volume	38.458	38.158	38.569	48.130
Distortion index (bond length)	0.05146	0.04429	0.04984	0.02627

Notes: (*a*) this work; (*b*) Fischer & Weiss (1973 ▶); (*c*) Bukvetskii *et al.* (1993 ▶); (*d*) Hitchman *et al.* (2002 ▶).

**Fig. 1.**
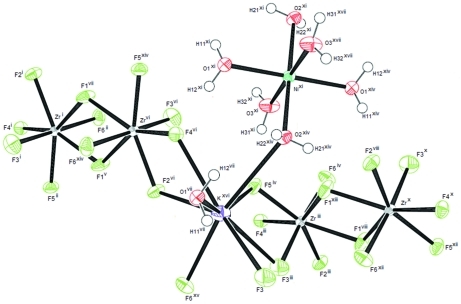
View of the polyhedral linkage inK2[Ni(H2O)6][ZrF6]2. Displacement ellipsoids are drawn at the 50% probability level. Symmetry codes: (i) x, y + 1, z; (ii) x - 1, y + 1, z; (iii) -x, -y + 1, -z + 1; (iv) -x, -y, -z; (v) -x, -y - 1, -z; (vi) -x - 1/2, y + 1/2, -z + 1/2; (vii) -x + 1/2, y + 1/2, -z + 1/2; (viii) x + 1/2, -y + 3/2, z - 1/2; (x) x + 1, y + 1, z; (xi) -x + 1/2, y - 1/2, -z + 1/2; (xii) x, y - 1, z; (xiii) x - 1, y - 1, z; (xiv) x + 1, y - 1, z; (xv) x - 1, y, z; (xvi) x - 1/2, -y + 3/2, z + 1/2; (xvii) -x - 1/2, y - 1/2, -z + 1/2.

**Fig. 2.**
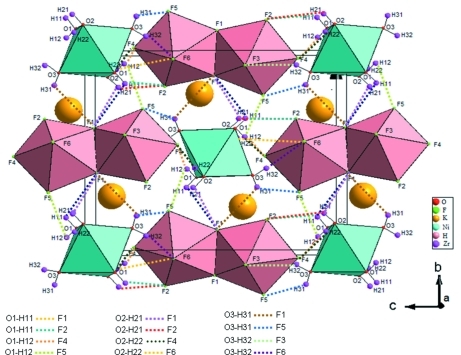
Projection of the crystal structure of K2[Ni(H2O)6][ZrF6]2 along [100] showing the hydrogen-bonding interactions.

## supplementary crystallographic information

### Comment

The existence of the title compound K_2_[Ni(H_2_O)_6_][ZrF_6_]_2_ as member of the large family of zirconium fluorido complexes with general formula *M*^I^_2_[*M*^II^(H_2_O)_6_][ZrF_6_]_2_ where *M*^I^ = K, Rb, Cs or NH_4_ and *M*^II^ = Co, Ni, Cu or Zn, has already been mentioned by Davidovich (1998). The monoclinic structure of the title compound is isotypic with those of K_2_[Cu(H_2_O)_6_][ZrF_6_]_2_ (Fischer & Weiss, 1973), K_2_[Zn(H_2_O)_6_][ZrF_6_]_2_ (Bukvetskii *et al.*, 1993) and Cs_2_[Zn(H_2_O)_6_][ZrF_6_]_2_ (Hitchman *et al.*, 2002). In the title structure the Ni^2+^ cation (site symmetry 1) is coordinated by six water molecules with two Ni—O distances slightly longer (2.0781 (9) Å) than the four others (2*x* 2.0548 (10) Å and 2*x* 2.0570 (11) Å). The Zr^4+^ cation is 7-coordinated by the fluoride ions but rather than being isolated anions, the fluoridozirconate(IV) ions form centrosymmetric [Zr_2_F_12_]^4-^ dimers. Thus the structure is built up from isolated and slightly elongated octahedral [Ni(H_2_O)_6_]^2+^ complex cations and dimeric [Zr_2_F_12_]^4-^ complex anions, also isolated from each other. The [ZrF_7_] coordination polyhedron is a distorted pentagonal bipyramid (symmetry 1) and the centrosymmetric [Zr_2_F_12_]^4-^ complex anion results from the association of two pentagonal bipyramids by sharing an equatorial edge F1—F1 passing through an inversion center of the unit cell corresponding to either the 2 *b* or 2 *c* Wyckoff positions. Both isolated [Ni(H_2_O)_6_]^2+^ and [Zr_2_F_12_]^4-^ complex ions lying in planes parallel to (010) are connected by the 8-coordinated K^+^ ions (Fig. 1) and an intricate O—H···F hydrogen bonds network (Fig. 2 and Table 1) to form the three-dimensional structure.

A careful examination of the geometry of the [Zr_2_F_12_]^4-^ complex anion in isotypic structures, refined from single-crystal data, shows this anion being quasi unvarying for all the members (Table 2). The distortion index (bond length) (Momma & Izumi, 2008) is the same for all the K compounds (0.0265) and is only very slightly higher (0.02985) for the Cs analogue. It is also worth noting that the higher the index of distortion of the [*M*^II^(H_2_O)_6_] cationic polyhedron, the lower the index of distortion of the counter cation K^+^ (0.04429). This observation is obvious because water molecules are only shared between K^+^ and *M*^2+^ ions.

### Experimental

Single crystals of the title compound were obtained by reacting a mixture of NiCl_2_.6H_2_O, ZrF_4_ and KCl in the molar ratio 1:2:2 with a 40% aqueous HF boiling solution in a platinum crucible. Then the solution was poured out into a PTFE beaker and slowly evaporated to dryness using a sand bath. Green single-crystals of the title compound were extracted from the dry residue.

### Refinement

The highest residual peak in the final difference Fourier map was located 0.60 Å from the Zr atom and the deepest hole was located 0.86 Å from the same atom. H atom parameter were fefined freely.

### Crystal data

**Table:**

K_2_[Ni(H_2_O)_6_][ZrF_6_]_2_	*F*(000) = 628
*M**_r_* = 655.45	*D*_x_ = 2.799 Mg m^−^^3^
Monoclinic, *P*2_1_/*n*	Mo *K*α radiation, λ = 0.71073 Å
Hall symbol: -P 2yn	Cell parameters from 7940 reflections
*a* = 6.6090 (1) Å	θ = 3.7–38.7°
*b* = 10.0398 (1) Å	µ = 3.20 mm^−^^1^
*c* = 11.7843 (1) Å	*T* = 296 K
β = 95.897 (1)°	Block, green
*V* = 777.79 (2) Å^3^	0.28 × 0.14 × 0.09 mm
*Z* = 2	

### Data collection

**Table:**

Bruker APEXII CCD diffractometer	4436 independent reflections
Radiation source: fine-focus sealed tube	3858 reflections with *I* > 2σ(*I*)
graphite	*R*_int_ = 0.028
Detector resolution: 8.3333 pixels mm^-1^	θ_max_ = 38.8°, θ_min_ = 4.2°
ω and φ scans	*h* = −11→11
Absorption correction: multi-scan (*SADABS*; Bruker, 2008)	*k* = −17→12
*T*_min_ = 0.613, *T*_max_ = 0.748	*l* = −18→20
17267 measured reflections	

### Refinement

**Table:**

Refinement on *F*^2^	Secondary atom site location: difference Fourier map
Least-squares matrix: full	Hydrogen site location: difference Fourier map
*R*[*F*^2^ > 2σ(*F*^2^)] = 0.023	All H-atom parameters refined
*wR*(*F*^2^) = 0.052	*w* = 1/[σ^2^(*F*_o_^2^) + (0.0182*P*)^2^ + 0.3179*P*] where *P* = (*F*_o_^2^ + 2*F*_c_^2^)/3
*S* = 1.06	(Δ/σ)_max_ = 0.032
4436 reflections	Δρ_max_ = 0.63 e Å^−^^3^
131 parameters	Δρ_min_ = −0.57 e Å^−^^3^
0 restraints	Extinction correction: *SHELXL97* (Sheldrick, 2008), Fc^*^=kFc[1+0.001xFc^2^λ^3^/sin(2θ)]^-1/4^
0 constraints	Extinction coefficient: 0.0068 (4)
Primary atom site location: structure-invariant direct methods	

### Special details

**Table:**

Geometry. All e.s.d.'s (except the e.s.d. in the dihedral angle between two l.s. planes) are estimated using the full covariance matrix. The cell e.s.d.'s are taken into account individually in the estimation of e.s.d.'s in distances, angles and torsion angles; correlations between e.s.d.'s in cell parameters are only used when they are defined by crystal symmetry. An approximate (isotropic) treatment of cell e.s.d.'s is used for estimating e.s.d.'s involving l.s. planes.
Refinement. Refinement of *F*^2^ against ALL reflections. The weighted *R*-factor *wR* and goodness of fit *S* are based on *F*^2^, conventional *R*-factors *R* are based on *F*, with *F* set to zero for negative *F*^2^. The threshold expression of *F*^2^ > σ(*F*^2^) is used only for calculating *R*-factors(gt) *etc*. and is not relevant to the choice of reflections for refinement. *R*-factors based on *F*^2^ are statistically about twice as large as those based on *F*, and *R*- factors based on ALL data will be even larger.

### Fractional atomic coordinates and isotropic or equivalent isotropic displacement parameters (Å^2^)

**Table:**

	*x*	*y*	*z*	*U*_iso_*/*U*_eq_	
Zr	−0.462200 (17)	0.984514 (11)	0.350424 (9)	0.01376 (3)	
Ni	0.0000	0.0000	0.0000	0.01508 (4)	
K	0.47534 (5)	0.71630 (3)	0.08423 (3)	0.02839 (7)	
O1	0.21314 (15)	−0.11683 (10)	−0.06747 (9)	0.02154 (17)	
O2	−0.23852 (15)	−0.12500 (10)	−0.05901 (9)	0.02039 (17)	
O3	−0.04437 (19)	−0.88871 (12)	−0.14701 (9)	0.0293 (2)	
F1	0.53736 (16)	0.11314 (9)	0.49592 (7)	0.0300 (2)	
F2	−0.53154 (14)	0.78950 (8)	0.31006 (7)	0.02440 (16)	
F3	−0.17901 (13)	0.93040 (11)	0.39692 (9)	0.0346 (2)	
F4	−0.46635 (13)	0.97177 (8)	0.17481 (7)	0.02228 (15)	
F5	−0.35001 (14)	0.16232 (8)	0.30407 (8)	0.02609 (17)	
F6	−0.75507 (12)	0.03609 (11)	0.31945 (8)	0.02759 (18)	
H11	0.159 (4)	−0.169 (3)	−0.111 (2)	0.044 (6)*	
H12	0.289 (4)	−0.072 (3)	−0.103 (2)	0.044 (6)*	
H21	−0.196 (3)	−0.178 (2)	−0.099 (2)	0.041 (6)*	
H22	−0.327 (3)	−0.084 (2)	−0.0981 (19)	0.038 (6)*	
H31	0.028 (4)	−0.829 (3)	−0.163 (2)	0.046 (7)*	
H32	−0.086 (4)	−0.925 (3)	−0.205 (2)	0.048 (7)*	

### Atomic displacement parameters (Å^2^)

**Table:**

	*U*^11^	*U*^22^	*U*^33^	*U*^12^	*U*^13^	*U*^23^
Zr	0.01662 (5)	0.01302 (5)	0.01144 (5)	−0.00068 (3)	0.00044 (3)	0.00045 (3)
Ni	0.01666 (9)	0.01442 (9)	0.01395 (9)	−0.00119 (6)	0.00064 (7)	−0.00028 (6)
K	0.03371 (15)	0.02741 (14)	0.02299 (14)	−0.00580 (11)	−0.00224 (11)	0.00113 (10)
O1	0.0229 (4)	0.0194 (4)	0.0230 (5)	−0.0021 (3)	0.0058 (4)	−0.0031 (3)
O2	0.0207 (4)	0.0186 (4)	0.0213 (4)	−0.0003 (3)	−0.0007 (3)	−0.0024 (3)
O3	0.0407 (6)	0.0279 (5)	0.0175 (5)	−0.0127 (4)	−0.0053 (4)	0.0056 (4)
F1	0.0579 (6)	0.0175 (4)	0.0158 (4)	−0.0105 (4)	0.0091 (4)	−0.0021 (3)
F2	0.0394 (5)	0.0162 (3)	0.0173 (4)	−0.0029 (3)	0.0014 (3)	−0.0013 (3)
F3	0.0216 (4)	0.0442 (6)	0.0365 (5)	0.0060 (4)	−0.0048 (3)	0.0052 (4)
F4	0.0280 (4)	0.0239 (4)	0.0152 (3)	−0.0041 (3)	0.0037 (3)	−0.0007 (3)
F5	0.0367 (4)	0.0181 (4)	0.0247 (4)	−0.0067 (3)	0.0091 (3)	0.0002 (3)
F6	0.0193 (4)	0.0350 (5)	0.0280 (5)	0.0055 (3)	0.0004 (3)	−0.0062 (3)

### Geometric parameters (Å, °)

**Table:**

Zr—F3	1.9717 (9)	K—F3^xi^	3.1685 (12)
Zr—F6^i^	2.0006 (8)	O1—K^xii^	2.8929 (11)
Zr—F5^i^	2.0293 (8)	O1—H11	0.79 (3)
Zr—F2	2.0554 (8)	O1—H12	0.82 (3)
Zr—F4	2.0708 (8)	O2—K^xiii^	3.1015 (11)
Zr—F1^ii^	2.1467 (8)	O2—H21	0.78 (2)
Zr—F1^iii^	2.1614 (8)	O2—H22	0.82 (2)
Ni—O1^iv^	2.0548 (10)	O3—Ni^xii^	2.0570 (11)
Ni—O1	2.0548 (10)	O3—H31	0.80 (3)
Ni—O3^v^	2.0570 (11)	O3—H32	0.80 (3)
Ni—O3^i^	2.0570 (11)	F1—Zr^xiv^	2.1467 (8)
Ni—O2^iv^	2.0781 (9)	F1—Zr^iii^	2.1614 (8)
Ni—O2	2.0781 (9)	F2—K^xv^	2.7658 (9)
K—F6^vi^	2.6497 (9)	F3—K^xvi^	2.7603 (10)
K—F5^vii^	2.7365 (10)	F3—K^vii^	3.1685 (12)
K—F3^viii^	2.7603 (10)	F4—K^xv^	2.7896 (9)
K—F2^ix^	2.7658 (9)	F5—Zr^xii^	2.0293 (8)
K—F4^ix^	2.7896 (9)	F5—K^xi^	2.7365 (10)
K—O1^i^	2.8929 (11)	F6—Zr^xii^	2.0006 (8)
K—O2^x^	3.1015 (11)	F6—K^xvii^	2.6497 (9)
			
F3—Zr—F6^i^	174.24 (4)	F5^vii^—K—F2^ix^	72.14 (3)
F3—Zr—F5^i^	87.40 (4)	F3^viii^—K—F2^ix^	150.45 (3)
F6^i^—Zr—F5^i^	95.54 (4)	F6^vi^—K—F4^ix^	121.62 (3)
F3—Zr—F2	89.11 (4)	F5^vii^—K—F4^ix^	85.15 (3)
F6^i^—Zr—F2	90.90 (4)	F3^viii^—K—F4^ix^	145.26 (3)
F5^i^—Zr—F2	148.71 (3)	F2^ix^—K—F4^ix^	53.33 (2)
F3—Zr—F4	100.10 (4)	F6^vi^—K—O1^i^	109.85 (3)
F6^i^—Zr—F4	85.44 (4)	F5^vii^—K—O1^i^	150.10 (3)
F5^i^—Zr—F4	75.70 (3)	F3^viii^—K—O1^i^	70.50 (3)
F2—Zr—F4	74.34 (3)	F2^ix^—K—O1^i^	111.88 (3)
F3—Zr—F1^ii^	91.31 (4)	F4^ix^—K—O1^i^	75.79 (3)
F6^i^—Zr—F1^ii^	84.80 (4)	F6^vi^—K—O2^x^	167.54 (3)
F5^i^—Zr—F1^ii^	73.52 (3)	F5^vii^—K—O2^x^	77.96 (3)
F2—Zr—F1^ii^	137.67 (3)	F3^viii^—K—O2^x^	92.00 (3)
F4—Zr—F1^ii^	146.55 (3)	F2^ix^—K—O2^x^	117.28 (3)
F3—Zr—F1^iii^	86.30 (4)	F4^ix^—K—O2^x^	70.61 (3)
F6^i^—Zr—F1^iii^	88.22 (4)	O1^i^—K—O2^x^	74.11 (3)
F5^i^—Zr—F1^iii^	138.27 (3)	F6^vi^—K—F3^xi^	66.39 (3)
F2—Zr—F1^iii^	72.35 (3)	F5^vii^—K—F3^xi^	55.25 (2)
F4—Zr—F1^iii^	145.96 (3)	F3^viii^—K—F3^xi^	72.07 (3)
F1^ii^—Zr—F1^iii^	65.45 (4)	F2^ix^—K—F3^xi^	102.83 (3)
F3—Zr—Zr^xviii^	88.57 (3)	F4^ix^—K—F3^xi^	139.92 (3)
F6^i^—Zr—Zr^xviii^	85.86 (3)	O1^i^—K—F3^xi^	142.29 (3)
F5^i^—Zr—Zr^xviii^	106.09 (3)	O2^x^—K—F3^xi^	103.01 (3)
F2—Zr—Zr^xviii^	104.90 (2)	F6^vi^—K—Zr^xi^	89.24 (2)
F4—Zr—Zr^xviii^	171.25 (2)	F5^vii^—K—Zr^xi^	28.097 (18)
F1^ii^—Zr—Zr^xviii^	32.85 (2)	F3^viii^—K—Zr^xi^	93.88 (2)
F1^iii^—Zr—Zr^xviii^	32.60 (2)	F2^ix^—K—Zr^xi^	93.56 (2)
F3—Zr—K^vii^	51.84 (3)	F4^ix^—K—Zr^xi^	112.72 (2)
F6^i^—Zr—K^vii^	129.16 (3)	O1^i^—K—Zr^xi^	151.51 (2)
F5^i^—Zr—K^vii^	39.43 (3)	O2^x^—K—Zr^xi^	83.09 (2)
F2—Zr—K^vii^	139.29 (3)	F3^xi^—K—Zr^xi^	29.294 (16)
F4—Zr—K^vii^	99.03 (2)	F6^vi^—K—Zr^ix^	97.85 (2)
F1^ii^—Zr—K^vii^	63.98 (3)	F5^vii^—K—Zr^ix^	75.428 (19)
F1^iii^—Zr—K^vii^	110.72 (3)	F3^viii^—K—Zr^ix^	163.11 (2)
Zr^xviii^—Zr—K^vii^	87.200 (6)	F2^ix^—K—Zr^ix^	26.431 (17)
F3—Zr—K^xv^	92.84 (3)	F4^ix^—K—Zr^ix^	27.023 (17)
F6^i^—Zr—K^xv^	90.62 (3)	O1^i^—K—Zr^ix^	95.67 (2)
F5^i^—Zr—K^xv^	112.37 (3)	O2^x^—K—Zr^ix^	93.42 (2)
F2—Zr—K^xv^	36.80 (2)	F3^xi^—K—Zr^ix^	121.99 (2)
F4—Zr—K^xv^	37.74 (2)	Zr^xi^—K—Zr^ix^	102.640 (7)
F1^ii^—Zr—K^xv^	172.94 (3)	F6^vi^—K—Zr^xvii^	20.660 (19)
F1^iii^—Zr—K^xv^	109.12 (2)	F5^vii^—K—Zr^xvii^	120.27 (2)
Zr^xviii^—Zr—K^xv^	141.547 (7)	F3^viii^—K—Zr^xvii^	67.44 (2)
K^vii^—Zr—K^xv^	123.004 (4)	F2^ix^—K—Zr^xvii^	83.04 (2)
F3—Zr—K^vi^	148.57 (3)	F4^ix^—K—Zr^xvii^	121.05 (2)
F6^i^—Zr—K^vi^	27.86 (3)	O1^i^—K—Zr^xvii^	89.46 (2)
F5^i^—Zr—K^vi^	83.54 (3)	O2^x^—K—Zr^xvii^	157.34 (2)
F2—Zr—K^vi^	113.62 (3)	F3^xi^—K—Zr^xvii^	80.348 (18)
F4—Zr—K^vi^	106.63 (3)	Zr^xi^—K—Zr^xvii^	106.804 (8)
F1^ii^—Zr—K^vi^	57.25 (3)	Zr^ix^—K—Zr^xvii^	103.843 (7)
F1^iii^—Zr—K^vi^	80.80 (3)	F6^vi^—K—Zr^viii^	65.81 (2)
Zr^xviii^—Zr—K^vi^	65.435 (5)	F5^vii^—K—Zr^viii^	137.16 (2)
K^vii^—Zr—K^vi^	106.804 (8)	F3^viii^—K—Zr^viii^	20.89 (2)
K^xv^—Zr—K^vi^	118.448 (4)	F2^ix^—K—Zr^viii^	131.81 (2)
F3—Zr—K^xvi^	29.95 (3)	F4^ix^—K—Zr^viii^	137.38 (2)
F6^i^—Zr—K^xvi^	144.59 (3)	O1^i^—K—Zr^viii^	63.49 (2)
F5^i^—Zr—K^xvi^	110.23 (3)	O2^x^—K—Zr^viii^	107.49 (2)
F2—Zr—K^xvi^	79.65 (3)	F3^xi^—K—Zr^viii^	82.684 (18)
F4—Zr—K^xvi^	123.50 (3)	Zr^xi^—K—Zr^viii^	109.132 (8)
F1^ii^—Zr—K^xvi^	79.97 (3)	Zr^ix^—K—Zr^viii^	143.549 (10)
F1^iii^—Zr—K^xvi^	56.37 (3)	Zr^xvii^—K—Zr^viii^	50.252 (5)
Zr^xviii^—Zr—K^xvi^	64.313 (5)	Ni—O1—K^xii^	118.84 (4)
K^vii^—Zr—K^xvi^	70.868 (8)	Ni—O1—H11	110.3 (17)
K^xv^—Zr—K^xvi^	100.955 (4)	K^xii^—O1—H11	102.4 (18)
K^vi^—Zr—K^xvi^	129.748 (5)	Ni—O1—H12	111.2 (17)
O1^iv^—Ni—O1	180.00 (7)	K^xii^—O1—H12	106.2 (17)
O1^iv^—Ni—O3^v^	91.62 (5)	H11—O1—H12	107 (2)
O1—Ni—O3^v^	88.38 (5)	Ni—O2—K^xiii^	127.74 (4)
O1^iv^—Ni—O3^i^	88.38 (5)	Ni—O2—H21	107.6 (17)
O1—Ni—O3^i^	91.62 (5)	K^xiii^—O2—H21	105.2 (17)
O3^v^—Ni—O3^i^	180.0	Ni—O2—H22	111.1 (16)
O1^iv^—Ni—O2^iv^	93.00 (4)	K^xiii^—O2—H22	97.1 (15)
O1—Ni—O2^iv^	87.00 (4)	H21—O2—H22	106 (2)
O3^v^—Ni—O2^iv^	90.50 (4)	Ni^xii^—O3—H31	124.4 (17)
O3^i^—Ni—O2^iv^	89.50 (4)	Ni^xii^—O3—H32	118.5 (19)
O1^iv^—Ni—O2	87.00 (4)	H31—O3—H32	108 (2)
O1—Ni—O2	93.00 (4)	Zr^xiv^—F1—Zr^iii^	114.55 (4)
O3^v^—Ni—O2	89.50 (4)	Zr—F2—K^xv^	116.77 (3)
O3^i^—Ni—O2	90.50 (4)	Zr—F3—K^xvi^	129.16 (5)
O2^iv^—Ni—O2	180.00 (5)	Zr—F3—K^vii^	98.87 (4)
F6^vi^—K—F5^vii^	99.70 (3)	K^xvi^—F3—K^vii^	107.93 (3)
F6^vi^—K—F3^viii^	78.70 (3)	Zr—F4—K^xv^	115.24 (3)
F5^vii^—K—F3^viii^	121.39 (3)	Zr^xii^—F5—K^xi^	112.48 (4)
F6^vi^—K—F2^ix^	72.85 (3)	Zr^xii^—F6—K^xvii^	131.48 (4)

Symmetry codes: (i) *x*, *y*+1, *z*; (ii) *x*−1, *y*+1, *z*; (iii) −*x*, −*y*+1, −*z*+1; (iv) −*x*, −*y*, −*z*; (v) −*x*, −*y*−1, −*z*; (vi) −*x*−1/2, *y*+1/2, −*z*+1/2; (vii) −*x*+1/2, *y*+1/2, −*z*+1/2; (viii) *x*+1/2, −*y*+3/2, *z*−1/2; (ix) *x*+1, *y*, *z*; (x) *x*+1, *y*+1, *z*; (xi) −*x*+1/2, *y*−1/2, −*z*+1/2; (xii) *x*, *y*−1, *z*; (xiii) *x*−1, *y*−1, *z*; (xiv) *x*+1, *y*−1, *z*; (xv) *x*−1, *y*, *z*; (xvi) *x*−1/2, −*y*+3/2, *z*+1/2; (xvii) −*x*−1/2, *y*−1/2, −*z*+1/2; (xviii) −*x*−1, −*y*+2, −*z*+1.

### Hydrogen-bond geometry (Å, °)

**Table:**

*D*—H···*A*	*D*—H	H···*A*	*D*···*A*	*D*—H···*A*
O1—H11···F1^xi^	0.79 (3)	2.94 (2)	3.3300 (14)	112.8 (19)
O1—H11···F2^xix^	0.79 (3)	1.92 (3)	2.6877 (14)	164 (2)
O1—H12···F4^xx^	0.82 (3)	1.82 (3)	2.6375 (13)	177 (2)
O1—H12···F5^iv^	0.82 (3)	2.61 (2)	3.0526 (13)	116 (2)
O2—H21···F1^xi^	0.78 (2)	2.59 (2)	3.0048 (13)	115 (2)
O2—H21···F2^xix^	0.78 (2)	1.95 (2)	2.7227 (13)	167 (2)
O2—H22···F4^xxi^	0.82 (2)	1.92 (2)	2.7362 (13)	173 (2)
O2—H22···F6^xxii^	0.82 (2)	2.76 (2)	3.1924 (14)	114.9 (17)
O3—H31···F1^xxiii^	0.80 (3)	2.86 (3)	3.2552 (16)	113.3 (19)
O3—H31···F5^xxiv^	0.80 (3)	1.92 (3)	2.7019 (14)	168 (2)
O3—H32···F3^iv^	0.80 (3)	3.00 (2)	3.4527 (16)	119 (2)
O3—H32···F6^xxv^	0.80 (3)	1.97 (3)	2.7427 (15)	163 (2)

Symmetry codes: (xi) −*x*+1/2, *y*−1/2, −*z*+1/2; (xix) *x*+1/2, −*y*+1/2, *z*−1/2; (xx) −*x*, −*y*+1, −*z*; (iv) −*x*, −*y*, −*z*; (xxi) −*x*−1, −*y*+1, −*z*; (xxii) −*x*−1, −*y*, −*z*; (xxiii) *x*−1/2, −*y*−1/2, *z*−1/2; (xxiv) *x*+1/2, −*y*−1/2, *z*−1/2; (xxv) −*x*−1, −*y*−1, −*z*.

### Table 2 Comparison of the geometrical characteristics of the coordination polyhedra in isotypic *M*^I^_2_[*M*^II^(H_2_O)_6_][ZrF_6_]_2_ structures determined from single-crystal data (Å, °, Å^3^)

**Table:**

	K_2_[Ni(H_2_O)_6_][ZrF_6_]_2_*^a^*	K_2_[Cu(H_2_O)_6_][ZrF_6_]_2_*^b^*	K_2_[Zn(H_2_O)_6_][ZrF_6_]_2_*^c^*	Cs_2_[Zn(H_2_O)_6_][ZrF_6_]_2_*^d^*
Space group	*P*2_1_/*n*	*P*2_1_/*c*	*P*2_1_/*c*	*P*2_1_/*n*
*a*	6.6090 (1)	6.631 (6)	6.631 (1)	6.970 (1)
*b*	10.0398 (1)	9.981 (10)	10.071 (1)	10.515 (2)
*c*	11.7843 (1)	12.921 (12)	12.952 (1)	11.803 (2)
*β*	95.897 (1)	114.20 (15)	114.96 (2)	93.56 (3)
*V*	777.786 (16)	780.01 (1)	784.16 (2)	863.4 (3)
	Ni—O1 = 2.0548 (10) (2×)	Cu—O1 = 1.966 (4) (2×)	Zn—O1 = 2.0856 (2) (2×)	Zn—O3 = 2.096 (6) (2×)
Distances *M*^II^—O	Ni—O3 = 2.0570 (11) (2×)	Cu—O2 = 2.025 (6) (2×)	Zn—O2 = 2.0940 (1) (2×)	Zn—O1 = 2.099 (5) (2×)
	Ni—O2 = 2.0781 (9) (2×)	Cu—O3 = 2.327 (5) (2×)	Zn—O3 = 2.1185 (2) (2×)	Zn—O2 = 2.105 (5) (2×)
Average *M*^II^—O bond length	2.063	2.106	2.099	2.100
Polyhedral volume	11.684	12.335	12.318	12.341
Distortion index (bond length)	0.00483	0.06089	0.00607	0.00156
Quadratic elongation	1.0013	1.0124	1.0011	1.0006
	Zr—F6 = 1.9718 (9)	Zr—F3 = 1.968 (5)	Zr—F3 = 1.9727 (3)	Zr—F3 = 1.962 (5)
	Zr—F5 = 2.0006 (8)	Zr—F1 = 2.004 (5)	Zr—F1 = 2.0018 (3)	Zr—F6 = 1.977 (5)
	Zr—F3 = 2.0293 (9)	Zr—F5 = 2.029 (4)	Zr—F5 = 2.0277 (3)	Zr—F5 = 2.037 (5)
Distances Zr—F (Å)	Zr—F2 = 2.0554 (8)	Zr—F6 = 2.059 (4)	Zr—F6 = 2.0570 (3)	Zr—F4 = 2.067 (4)
	Zr—F1 = 2.0708 (8)	Zr—F2 = 2.063 (4)	Zr—F2 = 2.0668 (4)	Zr—F2 = 2.069 (4)
	Zr—F4 = 2.1468 (9)	Zr—F4 = 2.156 (5)	Zr—F4 = 2.1501 (4)	Zr—F1 = 2.156 (4)
	Zr—F4 = 2.1614 (8)	Zr—F4 = 2.160 (4)	Zr—F4 = 2.1628 (4)	Zr—F1 = 2.180 (4)
Average Zr—F bond length	2.062	2.063	2.063	2.064
Polyhedral volume	13.669	13.674	13.675	13.692
Distortion index (bond length)	0.02662	0.02650	0.02654	0.02985
	K—F5 = 2.6496 (10)	K—F1 = 2.668 (5)	K—F1 = 2.6506 (3)	Cs—F6 = 2.911 (5)
	K—F3 = 2.7366 (9)	K—F6 = 2.750 (6)	K—F2 = 2.7395 (4)	Cs—F4 = 3.046 (4)
	K—F6 = 2.7603 (11)	K—F3 = 2.756 (5)	K—F5 = 2.7633 (2)	Cs—F2 = 3.057 (4)
Distances K—F/O	K—F2 = 2.7658 (9)	K—F5 = 2.767 (5)	K—F6 = 2.7739 (2)	Cs—F3 = 3.065 (5)
	K—F1 = 2.7895 (9)	K—F2 = 2.799 (5)	K—F3 = 2.8094 (2)	Cs—F3 = 3.102 (5)
	K—O2 = 2.8927 (10)	K—O3 = 2.942 (5)	K—O1 = 2.8968 (3)	Cs—O2 = 3.218 (5)
	K—O1 = 3.1012 (10)	K—O1 = 2.980 (5)	K—O2 = 3.0873 (5)	Cs—O1 = 3.236 (5)
	K—F6 = 3.1684 (12)	K—F3 = 3.1307 (7)	K—F3 = 3.1707 (7)	Cs—F5 = 3.228 (6)
Average *M*^I^—F/O bond length	2.858	2.849	2.861	3.118
Polyhedral volume	38.458	38.158	38.569	48.130
Distortion index (bond length)	0.05146	0.04429	0.04984	0.02627

Notes: (*a*) this work; (*b*) Fischer & Weiss (1973); (*c*) Bukvetskii *et al.* (1993); (*d*) Hitchman *et al.* (2002).

## References

[bb1] Brandenburg, K. (1999). *DIAMOND* Crystal Impact GbR, Bonn, Germany.

[bb2] Bruker (2008). *SADABS*, *APEX2* and *SAINT* Bruker AXS Inc., Madison, Wisconsin, USA.

[bb3] Bukvetskii, B. V., Gerasimenko, A. V., Davidovich, R. L., Kaidalova, T. A. & Teplukhina, L. V. (1993). *Koord. Khim.* **19**, 526–528.

[bb4] Davidovich, R. L. (1998). *Russ. J. Coord. Chem.* **24**, 751–768.

[bb5] Farrugia, L. J. (1997). *J. Appl. Cryst.* **30**, 565.

[bb6] Fischer, J. & Weiss, R. (1973). *Acta Cryst.* B**29**, 1958–1962.

[bb7] Hitchman, M. A., Yablokov, Yu. V., Petrashen, V. E., Augustyniak-Jublokov, M. A., Stratemeier, H., Riley, M. J., Lukaszewicz, K., Tomaszewski, P. E. & Pietraszko, A. (2002). *Inorg. Chem.* **41**, 229–238. 10.1021/ic010464d 11800611

[bb8] Momma, K. & Izumi, F. (2008). *J. Appl. Cryst.* **41**, 653–658.

[bb9] Sheldrick, G. M. (2008). *Acta Cryst.* A**64**, 112–122. 10.1107/S0108767307043930 18156677

